# Magnesium in Aging, Health and Diseases

**DOI:** 10.3390/nu13020463

**Published:** 2021-01-30

**Authors:** Mario Barbagallo, Nicola Veronese, Ligia J. Dominguez

**Affiliations:** Geriatric Unit, Department of Medicine, University of Palermo, 90127 Palermo, Italy; nicola.veronese@unipa.it (N.V.); ligia.dominguez@unipa.it (L.J.D.)

**Keywords:** magnesium, oxidative stress, diseases, dementia, diabetes, osteoporosis, aging, hypertension, health, longevity

## Abstract

Several changes of magnesium (Mg) metabolism have been reported with aging, including diminished Mg intake, impaired intestinal Mg absorption and renal Mg wasting. Mild Mg deficits are generally asymptomatic and clinical signs are usually non-specific or absent. Asthenia, sleep disorders, hyperemotionality, and cognitive disorders are common in the elderly with mild Mg deficit, and may be often confused with age-related symptoms. Chronic Mg deficits increase the production of free radicals which have been implicated in the development of several chronic age-related disorders. Numerous human diseases have been associated with Mg deficits, including cardiovascular diseases, hypertension and stroke, cardio-metabolic syndrome and type 2 diabetes mellitus, airways constrictive syndromes and asthma, depression, stress-related conditions and psychiatric disorders, Alzheimer’s disease (AD) and other dementia syndromes, muscular diseases (muscle pain, chronic fatigue, and fibromyalgia), bone fragility, and cancer. Dietary Mg and/or Mg consumed in drinking water (generally more bioavailable than Mg contained in food) or in alternative Mg supplements should be taken into consideration in the correction of Mg deficits. Maintaining an optimal Mg balance all through life may help in the prevention of oxidative stress and chronic conditions associated with aging. This needs to be demonstrated by future studies.

## 1. Introduction

Magnesium ion (Mg) is the divalent intracellular cation most present in the human cell and the second cation after potassium (K). Mg atomic weight is 24.305 g/mol, and its atomic number is 12 (Table 1). Mg has a crucial role in numerous biological processes, including oxidative phosphorylation, energy production, glycolysis, protein and nucleic acid synthesis [1]. Mg plays a role in the mitochondrial synthesis of adenosine triphosphate (ATP) to form MgATP [2]. Cell signaling needs MgATP for protein phosphorylation and activation of cyclic adenosine monophosphate (cAMP), which is involved in a number of biochemical processes [3]. Mg ions participate in the transport of other ions through cell membranes, in muscle contraction, and in controlling neuron excitability. Cellular Mg homeostasis is linked to the cellular metabolism of other ions, i.e., K, sodium (Na), calcium (Ca), via Na^+^/K^+^/ATPase, Ca^++^ activated K channels, and other mechanisms [4].

Mg has a key role for cellular homeostasis and organ functioning. Thus, Mg has a physiological role in controlling various key cellular activities and metabolic pathways, including enzyme substrate, structural and membrane functions [2,5]. Mg is a cofactor in over 600 enzymatic reactions and is required for the activity of protein kinases, glycolytic enzymes, for all phosphorylation processes, and for all reactions that implicate ATP [2,5]. Mg ion has a mild Ca antagonist action and is involved in a number of structural functions (multi-enzyme complexes, i.e., G-proteins, proteins and nucleic acids synthesis, N-methyl-D-aspartic acid (NMDA) receptors, mitochondria, polyribosomes, etc.).

In the last decades, the pathophysiological and clinical importance of Mg has been acknowledged, as well as the possible effects of Mg deficits on several human diseases.

## 2. Mg Metabolism and Requirement

**Figure 1:**
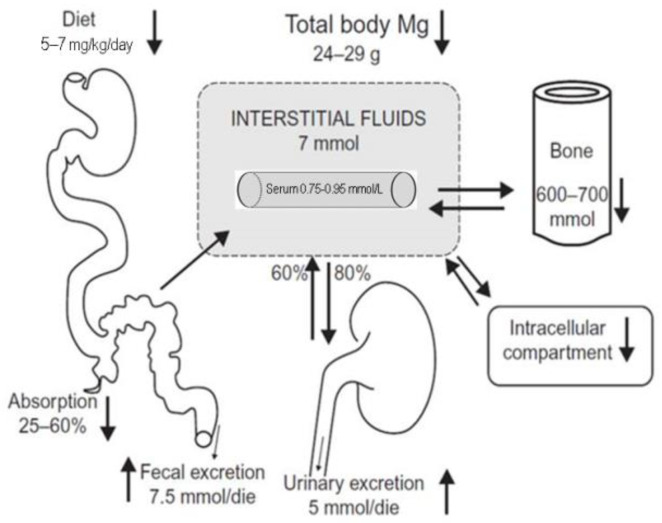
Mg balance (arrows show most common sites of Mg depletion with aging), including daily amount of Mg intake and excretion. Total human body content of Mg is 24 to 29 g. In order to maintain Mg balance, a healthy person needs to consume around 5–7 mg/kg/day. Daily intestinal absorption varies from 25 to 60% of Mg intake. In the kidney, 80% of circulating Mg is filtered and about 60% is reabsorbed along the kidney tubule. This results in a net excretion of about 5 mmol/day. Fecal excretion is about 7.5 mmol/day. The intracellular compartment provides the most important Mg stores.

The Mg content in the human body is around 24–29 g of Mg, of which near 2/3 are deposited in bone and 1/3 in the cells. Only <1% of the total Mg is extracellular. Mg levels in the serum range between 0.75 and 0.95 mmol/L. Serum Mg levels in healthy subjects are very constant and tightly preserved within this narrow range by a dynamic balance among Mg intake, its intestinal absorption, kidney excretion, the bone storage and the Mg requirement of different tissues. Mg absorption is increased under conditions of Mg limited assumption. If Mg deprivation persists, bone storages would help to preserve serum Mg levels by replacing part of its content in the extracellular compartment [6] (Figure 1). Serum Mg levels are considered low if inferior than 0.75 mmol/L, while frank hypomagnesemia is generally considered as serum Mg level lower than 0.7 mmol/L [1,2,7]. Total serum Mg levels (MgT) are not a sufficiently precise measure of the body Mg status; MgT levels are more useful in epidemiological studies, but are not enough accurate to detect subclinical Mg deficits on a single subject [8]. This is because serum total Mg levels do not accurately mirror intracellular concentrations, and low intracellular Mg levels generally precede alterations of serum Mg. It is thus possible to have intracellular and storage Mg depletion with still normal total serum Mg values [6]. 

Optimal Mg requirement with food is considered to be 320 mg/day for women and 420 mg/day for men, according to the 2015–2020 Dietary Guidelines for Americans [9], but higher requirements may be needed in some physiologic conditions such as pregnancy, aging, or during exercise and in some pathological conditions (i.e., infections, type 2 diabetes mellitus (T2DM), etc.).

Many factors may alter Mg balance: a high content in the diet of Na, Ca, protein, alcohol or caffeine, or the use of certain drugs (diuretics, e.g., furosemide; proton-pump inhibitors, e.g., omeprazole, etc.). Mg absorption mainly occurs in the small intestine [10]. To maintain the balance, a healthy person needs to consume around 5–7 mg/kg/day (Table 2). Mg deposited in the bone is not easily exchanged and any rapid Mg requirement is provided by the Mg present in the intracellular compartment. The kidney helps to control and modulate Mg balance; each day around 120 mg of Mg is eliminated into the urine [1]. Renal Mg control is strictly dependent on Mg status, since Mg depletion stimulates Mg reabsorption across the nephron, while Mg urinary excretion is decreased in conditions of body Mg depletion [11]. Diuretics alter renal Mg handling increasing Mg wasting [12]. No hormone is known to be a specific Mg regulator. However, many hormonal factors have an identified effect on Mg homeostasis (i.e., insulin, parathyroid hormone (PTH), calcitonin, catecholamines) [6,13]. 

## 3. Mg Deficiencies Associated with Reduced Mg Intake

Several studies have consistently shown that in western countries the average dietary Mg intakes are often inadequate [14] and significantly lower than the recommended daily intake of Mg [15]. King et al. in 2005 reported that almost 2/3 of the Americans have a Mg consumption below the recommended daily allowance (RDA). In forty-five percent of subjects, the daily intake was less than seventy-five percent of the RDA, and in nineteen percent the daily intake was less than fifty percent of the RDA [16]. In Europe, the situation is similar [15]. Even in physically active and well-educated European women, dietary recommendations are not followed [17]. Western diets are generally rich in refined foods that are very poor in Mg while also having a very low content of whole grains and green vegetables, which are foods rich in Mg content. Cooking and the refining processes may consistently diminish the Mg content present in the food since a significant amount of Mg is lost during these procedures. Thus, diets rich in refined or processed foods are likely to be low in Mg. In particular, the boiling of foods is a major cause of loss of Mg [18]. The presence of a large quantity of refined and processed food in the western diets may help to explain the large percentage of individuals with a condition of Mg deficiency [19]. 

Pathogenic gut microbiota may alter Mg absorption from the diet. In ruminant, bacteria convert trans-aconitate to tricarballylate, a tricarboxylic acid which chelates blood divalent cations, such as Mg, and decreases their availability. Tricarballylate has been proposed as a factor involved in the hypomagnesemia that leads to grass tetany [20,21]. 

In addition, phytic acid present in some foods may lower Mg absorption. Glyphosate, a pesticide frequently used in the crops, may chelate minerals including Mg [22], further reducing the content of Mg in the soil and in some crops. Organic food, from pesticide-free soils, was shown to have significantly higher Mg content than non-organic control food [23]. 

Mg is widely used in many food products, including a variety of confectionery, spices and baking ingredients, and oral pharmaceutical formulations as anticaking agent, and in preventing contaminations in food and beverages [24]. 

Mg consumption from water rich in Mg may be taken into consideration as an alternative source of Mg [25]. Drinking water, especially mineral-rich harder water, can be rich in Mg salts; therefore, water may provide an important supplementary contribution to total Mg intake, representing a possible alternative to oral supplements, although the Ca/Mg ratio in water may play a role. In the SU.VI.MAX cohort, subjects who used to drink water rich in Mg resulted to have significantly higher Mg intakes than those who used to drink low mineralized or tap water [25]. 

The bio-availability of Mg in drinking water is generally higher when compared to Mg in food and while it is easy to add Mg to water, it is virtually impossible to add Mg to foods. 

The water content of Mg may be significant not only in the water used for drinking, but also in water used for cooking, since a higher concentration of Mg in the water used for boiling may reduce the leakage of Mg in food during cooking, and may reduce the loss of Mg in the boiled food. With increasing shortage of fresh water globally, the use of desalinated seawater (DSW) is becoming very common in many areas in the world [26]. In Israel, >50% of drinking water is now derived from DSW. Desalination removes Mg, and hypomagnesaemia has been associated with increased cardiac morbidity and mortality [27]. 

## 4. Mg Deficits Associated with Aging

Aging is often associated with a total body Mg deficit [19]. Serum Mg levels remain constant with age [28]. Alterations in serum Mg are usually associated with the existence of diseases and/or alterations in kidney function. In healthy older persons, an age-dependent decrease in cellular Mg concentration was previously shown [29] in the absence of alterations of total serum Mg. It has been confirmed that chronic latent Mg deficiency is quite common in older adults in western countries. Possible mechanisms of this demonstrated Mg insufficiency with aging are detailed in Table 3. This Mg shortage is frequently associated with a low Mg intake [30,31], while Mg requirements for the body processes do not change with age [32]. 

Data from the National Health and Nutrition Examination (NHANES) III have confirmed that aging is an additional risk factor for inadequate Mg consumptions and progressive decrease with age [30]. 

Intestinal absorption of Mg tends to fall with age, and this decline may be one of the possible causes of Mg deficit with aging [33]. The alteration of the intestinal absorption of Mg in old age is often worsened by impairment of vitamin D homeostasis, common in old age. Renal reabsorption of Mg is an active process occurring in the loop of Henle and in the proximal convoluted tubule. Reduced kidney functionality, common in the elderly, is a possible additional cause of Mg loss. 

Secondary Mg deficiencies in older adults may be linked to the presence of several conditions and related polypharmacotherapy [34]. Diuretic therapy may cause excessive Mg urinary loss. Diuretic-induced hypomagnesemia is often accompanied by hypokalemia. Hypomagnesemia may be present in around 40% of patients with hypokalemia, and the correction of Mg deficit is needed to achieve the correction of the K deficits. It is thus advisable to evaluate Mg levels in patients with hypokalemia. Other medications commonly used in the elderly may contribute to Mg deficits (e.g., antacids, H2 blockers, proton pump inhibitors, antihistamines, antibiotics, antiepileptic drugs, and antivirals, among others).

## 5. Mg, Inflammation and Oxidative Stress

Mg deprivation, low serum Mg levels, and reduced dietary Mg intake have all been associated in preclinical, epidemiological and clinical human studies with an increased production of free radicals of oxygen, with low-grade systemic inflammation, increased levels of inflammation markers and proinflammatory molecules (IL-6, tumor necrosis factor-alpha (TNF-alfa), IL-1-beta, vascular cell adhesion molecule (VCAM)-1, and plasminogen activator inhibitor (PAI)-1, complement, alfa2-macroblobulin, fibrinogen) [16,35,36,37,38,39,40,41,42].

King et al., using the NHANES database, found that dietary Mg intake was inversely related to reactive protein C levels [16]. Similar results were found by Song et al. using data from the Women’s Health Study in adult women [42]. 

Mg depletion results in an increased production of oxygen-derived free radicals (ROS) increased oxygen peroxide production, and increased production of superoxide anion by inflammatory cells. Mg deficiency not only increases the oxidative stress but also decreases the antioxidant defense competence [35,43]. Mg is required for the proper functioning of gamma-glutamyl transpeptidase, which plays a key role in the synthesis of antioxidant glutathione [44], confirming that Mg may have a mild antioxidant action [45].

In humans, a correlation between intracellular Mg and circulating reduced/oxidized glutathione ratio has been reported [46]. In another study, a negative correlation between Mg levels and oxidative stress markers (plasma superoxide anions and malondialdehyde) was detected in a population exposed to chronic stress [47].

**Figure 2:**
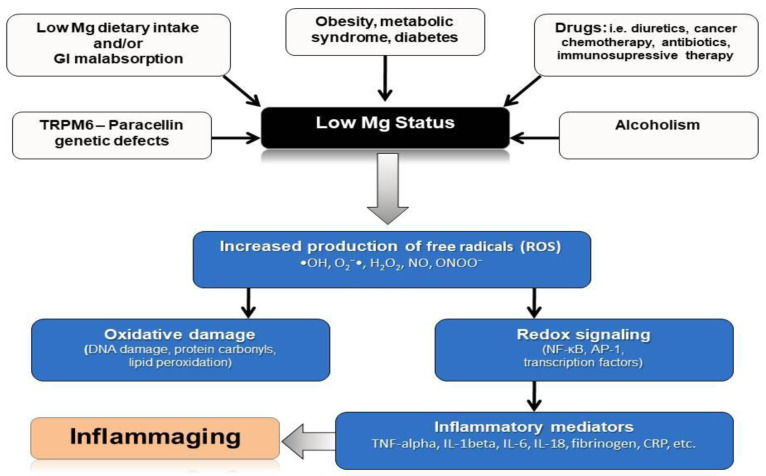
Mg deficit, inflammation, oxidative stress, and aging. The relationship of low Mg status, generated by multiple factors (i.e., low Mg intake and absorption, Mg transport genetic defects, obesity, type 2 diabetes mellitus (T2DM) and cardio-metabolic syndrome, polypharmacotherapy, and alcohol abuse), which may trigger an increased production of free radicals (ROS), oxidative damage, and activation of redox signaling (i.e., NF-KB, AP-1, and other transcription factors). The elevation in oxidative stress may lead to the release of inflammatory mediators conforming a state of chronic low-grade inflammation, which has been proposed to accompany aging and called “inflammaging”. TRPM7: Transient Receptor Potential cation channel, subfamily M, member 7; ROS: reactive oxygen species; NF-KB nuclear factor kappa-light-chain-enhancer of activated B cells; AP-1: activator protein 1; TNF-alpha: tumor necrosis factor-alpha; IL: interleukin; CRP: C-reactive protein.

Aging is accompanied by a low-grade inflammatory state that has been named “inflammaging” [48]. We have previously postulated that a chronic Mg insufficiency facilitating this inflammaging condition and an impairment of the redox status may facilitate the development of age-related illnesses (Figure 2) [19,49]. In particular, we have suggested a link between the Mg inadequacy and the occurrence of an insulin resistance state, T2DM, and cardiometabolic syndrome [2].

## 6. Mg and the Immune Responses

Mg modulates both innate and acquired immune responses and acts as a mediator in the signaling pathways controlling immune cell development, homeostasis, and activation [35]. Mg is a crucial cofactor for T helper-beta cell adherence, immunoglobulin synthesis, antibody-dependent cytolysis, IgM lymphocyte binding, and macrophage response to lymphokines [37,50]. Mg influences acquired immunity by modulating the proliferation and development of lymphocytes [51]. Transient Receptor Potential cation channel, subfamily M, member 7 (TRPM7) is crucial for Mg homeostasis in immune cells. A fall in free cytosolic Mg and cell cycle arrest was found in TRPM7-deficient B cell lines, which was preserved by culturing the cells in a medium containing high Mg. An impaired development of T cells was observed in TRPM7 knockout mice [52]. 

Mg deficiency may accelerate thymus involution. In thymuses from Mg-deficient rats, higher levels of apoptosis were observed, as compared with controls [53]. A Mg-deficient diet caused an alteration of polymorphonuclear cell number and functionality and an activation of phagocytosis [38]. Mg is involved in the regulation of cell apoptosis. Fas-induced β-cell apoptosis also requires Mg. A raise in cellular free Mg levels is necessary for the expression of Fas molecule binding on the β-cell surface to trigger signaling pathways that initiate cellular apoptosis and death [54]. In addition, Mg is involved in the synthesis, transport and activation of vitamin D, which is an important immunomodulator in several infectious diseases, including SARS-Cov2 infection [37]. Mg deficit may also be involved in other mechanisms described in COVID-19, such as the immune hyperresponsiveness with excessive release of inflammatory mediators leading to the cytokine storm, endothelial dysfunction, thrombotic complications, and pre-existent predisposing conditions that worsen the prognosis of COVID-19 clinical course, such as old age, diabetes and hypertension (see below). 

## 7. Clinical Symptoms Associated with Mg Deficits

Clinical signs and symptoms are generally absent or non-specific in moderate Mg deficits and mild hypomagnesemic subjects are usually asymptomatic. Non-specific manifestations may include anxiety, insomnia, fatigue, hyperemotionality, depressive symptoms, headache, light-headedness, dizziness. Most of these symptoms are non-specific and common in older patients, and might be mistaken with normal age-related manifestations. Other symptoms may be associated, such as myalgias, acroparesthesias, and cramps. Other non-specific functional complaints may include chest pain, *sine materia* dyspnea, precordialgia, palpitations, extrasystoles and other arrhythmias, etc. [55]. 

Several signs and symptoms are connected with severe Mg deficits including weakness, tremor, muscle fasciculation, dysphagia, presence of Chvostek’s sign (facial twitching as a reaction to the tapping of the facial nerve), or Trousseau’s sign (spasm of muscles of the hand and forearm following the application of a pressure cuff, to transiently occlude the brachial artery), orthostatic hypotension and/or borderline hypertension [55]. 

Elin suggested naming the condition of subjects with this non-specific symptomatology associated with chronic, negative Mg balance as a syndrome of “Chronic Latent Mg Deficiency” (CLMD) [8]. Subjects affected by CLMD generally present lower normal total serum Mg levels (latent) and are generally clinically undiagnosed, not having hypomagnesemia, but may benefit from Mg supplementation. 

## 8. Hypothesis on the Possible Role of Mg in the Aging Process and Longevity

Mg is crucial to preserve genomic stability in cellular systems, because of its stabilizing effects on DNA and chromatin structures. Thus, Mg ion is needed in nucleotide excision repair, base excision repair, and mismatch repair, and is crucial for the removal of DNA damage caused by endogenous processes, environmental mutagens, and DNA replication [56]. Mg deficiency triggers the cell vulnerability to oxidation and may affect immune system performance; a lack of Mg would alter membrane integrity and functionality and may facilitate mitochondrial alterations (decreased number, morphology modifications, increased apoptosis, increased DNA mutations, decreased biogenesis, decreased autophagy) [19]. Mg has an important role in the modulation of protein synthesis and membrane repair [56,57]. DNA is incessantly altered by endogenous processes and environmental mutagens. An increase in cellular Mg has been demonstrated in the early stages of apoptosis, possibly linked to a mobilization of Mg from the mitochondria; Mg may act as a “second messenger” for downstream events in apoptosis [54]. Mg deprivation increases the susceptibility to oxidative damage facilitating alterations of the membrane integrity and function. 

Some alterations in cell physiology occurring in senescence in different cell types [58] are similar to those caused by Mg deficit. Mg-depletion-related alterations include reduced protection from oxidative stress damage, reduced cell cycle progression, reduced culture growth, and reduced cellular viability as well as triggering of the expression of proto-oncogene and of transcription factors [59]. Culturing primary fibroblasts in Mg-deficient media would lower the replicative capacity and accelerate the expression of biomarkers associated with senescence and in telomere attrition. A decreased replicative lifespan was observed vs. fibroblast cultured in normal Mg media conditions [60]. 

Because of the essential role of Mg in the stabilization of DNA, in defending the cell to the damage of ROS and in stimulating DNA replication and transcription, a Mg deficit may facilitate genomic instability, alter DNA repair, and reduce mitochondria functionality, thus facilitating an accelerated cellular senescence and aging [56,61]. Mg has a demonstrated protective role against these effects and contrasts the shortening of telomeres (that is associated with aging and a reduced life expectancy), which is observed in low Mg conditions. It has been suggested that because of this action to prevent telomere shortening, correcting Mg deficiencies may prolong life [62]. 

## 9. Mg in Hypertension and Cardiovascular Diseases

In the last decades, Mg deficit has been connected with several cardiovascular conditions [2,63]. Kobayashi first noted in 1957 that the mineral composition of drinking water was associated with cardiovascular death rates; the stroke incidence was lower in regions with hard water (mainly linked to Mg and Ca content) [64]. Schroeder confirmed these data only a few years later, analyzing the relationship between water hardness and death rates. He found that cardiovascular death rates were significantly lower in states with hard water compared to states with soft water [65].

Mg is involved in blood pressure homeostasis. Although Mg has not a direct role in the biochemical mechanisms of contraction, classic studies from Altura et al. have demonstrated that Mg controls vascular tone and contractility by altering Ca levels, and that changes in Mg concentration modulates Ca-triggered vascular smooth muscle contraction [66,67,68]. Mg itself functions as nature’s weak physiologic Ca channel blocker [69], modulating Ca-channel activity in heart cells [70]. Mg deficit stimulates angiotensin II-mediated aldosterone synthesis, as well as thromboxane and vasoconstrictor prostaglandins production [71]. Mg has a favorable action on vascular endothelium modulating the release of nitric oxide, prostacyclin, and endothelin-1 [72]. In humans, oral Mg supplementation was shown to improve endothelial function in T2DM older adults [73]. 

Mg ion, because of these actions on vascular smooth muscle tone, plays a modulatory action on blood pressure homeostasis, while Mg deficits may be relevant to the physiopathology of hypertensive disorders. Altura showed that the depletion of Mg would induce vascular hyper-reactivity and elevation of blood pressure [74]. Serum total Mg levels are usually normal in hypertensive subjects. However, numerous defects of Mg homeostasis have been documented in hypertension. Former epidemiologic studies already showed an inverse relationship between Mg dietary intake and blood pressure [75]. In older populations, the age-related increase in blood pressure was concomitant with a reciprocal suppression of intracellular free Mg [29,76], suggesting a possible role for Mg deficit in the age-related elevation of blood pressure. Intracellular free Mg concentrations were found to be significantly lower in hypertensive subjects vs. normotensive controls [77]. Mg urinary excretion was also found to be altered in an experimental model of hypertensive rats [78]. High salt diet was reported to elevate blood pressure in salt-sensitive subjects, reciprocally suppressing intracellular free Mg levels [79]. 

Blackfan and Hamilton, as early as 1925, recommended Mg therapy to lower blood pressure in patients with malignant hypertension [80]. Intravenous (i.v.) Mg has been commonly used with consistent benefit in preeclampsia and in eclampsia [81], and in malignant hypertension [82]. However, the response to Mg oral supplement in essential hypertension is less clear [63,83,84,85,86]. In experimental hypertension, high or low Mg diets that, respectively, raised or reduced cellular free Mg, in parallel reduced and raised blood pressure. In humans, in some studies, Mg supplementation was found to have hypotensive effects, while in others it had no effect on blood pressure or may even worsen it [83,86]. Nevertheless, when quality studies are analyzed together in meta-analysis and systematic reviews the evidence is convincing, validating the key role of Mg in hypertension and of an inverse relationship of dietary Mg intake with the prevalence and incidence of hypertension [63]. However, a possible confounding factor of the evidence derived from observational studies is that diets containing elevated Mg intake are also usually low in Na and rich in K and of other elements with health benefits and thus high Mg intake may be, at least in part, a marker of a healthy diet [63].

Thus, a reproducible and persistent hypotensive effect of oral Mg supplements on blood pressure has not been yet confirmed in large long-term prospective trials in essential hypertension and further data are necessary to advise Mg supplementation as a non-pharmacological strategy for hypertension prevention and/or treatment purposes. There are several possible explanations for this uncertainty. Among them: (a) the virtual absence of specifically planned clinical prospective trials of Mg treatment in hypertension; (b) the different dosages and therapy schedules used in a number of smaller clinical reports; and (c) a failure to detect the heterogeneity of the underlying mechanisms causing the raise in blood pressure. Hence, long-term longitudinal therapeutic trials of oral Mg supplements in essential hypertension are required. 

Mg deficit has been linked to the development of atherosclerosis. Low Mg status may trigger vascular calcification, alter lipid metabolism and facilitate lipid accumulation in vascular plaques [87]. Serum Mg was found to be positively [88] or negatively [89] associated with serum lipid levels. Mg supplementation has been suggested to improve lipid profiles, and prevent atherosclerotic plaque formation and to act as a weak inhibitor of hydroxyl-3-methylglutaryl-coenzyme A reductase, and of other enzymes of the lipid metabolism [90]. 

Mg is involved in the heart’s electrical conduction and hypomagnesemia, hypokalemia and other electrolyte disturbances may trigger cardiac arrhythmias. Mg and K depletion also increase the susceptibility to arrhythmogenic effects of cardiac glycosides. 

Mg effects on conduction comprise prolongation of atrial and atrioventricular nodal refractory periods, which may help in rate and rhythm control in atrial fibrillation (AF) [91]. Mg supplements may be used as a supportive non-pharmacological treatment for atrial and/or ventricular arrhythmias [92]. AF may be associated with hypomagnesemia [93] and reduced serum Mg concentration may facilitate the development of AF [94]. 

In postmenopausal women, dietary Mg deficiency was associated with heart rhythm abnormalities, including AF and flutter that may respond to Mg supplementation [95]. A meta-analysis has suggested that i.v. Mg administration may have a role in the acute management of AF [96]. Because of its rapid, effective and simple application, i.v. Mg administration has been indicated in the treatment of torsade de pointes [97,98]. Antiarrhythmic actions of elevated Mg dietary intake have been suggested to mediate the reduced risk of sudden death in women in the highest quartile of Mg intake [99].

Oral Mg supplements were suggested to help improving clinical symptoms and survival outcomes vs. placebo in patients with severe congestive heart failure [100]. 

A systematic review and meta-analysis of prospective studies that included over 300,000 individuals found that elevated Mg serum levels paralleled a reduced risk of cardiovascular disease; elevated dietary Mg intakes were shown to be inversely associated with ischemic heart disease [101]. 

Another meta-analysis of prospective trials including a total of 241,378 subjects reported an inverse association between Mg intake and the risk of stroke [102]. A recent umbrella review evaluating health outcomes connected with Mg intake and supplementation confirmed the link between higher Mg intake and decreased risk of stroke [86].

Mg sulfate has been found to be protective both in preclinical models of stroke and in humans. Mg has been suggested to have some potential efficacy, and a good safety profile if delivered i.v. early after stroke onset [103]. 

## 10. Mg in Type 2 Diabetes 

A consistent body of evidence has linked Mg deficiency to alterations of insulin sensitivity and T2DM. Indeed, T2DM has been associated with several extra-and intra-cellular Mg abnormalities [2,104,105,106]. Lower cellular and/or ionized plasma Mg concentrations have been found in patients with T2DM despite still normal less sensitive total serum Mg levels [107,108]. Possible mechanisms favoring Mg depletion in T2DM include a low Mg dietary intake and an increased Mg urinary loss, while the absorption and retention of dietary Mg seem to be unchanged [109]. It has been reported an inverse relationship between Mg intake and the incidence of new cases of T2DM. Both hyperglycemia and hyperinsulinemia have been implicated in contributing to Mg depletion [110]. Both, hyperglycemia and hyperinsulinemia favor an excessive urinary Mg excretion, while insulin resistance may alter Mg transport [111]. Altered Mg metabolism may predispose to the development of T2DM and to an impairment of insulin-mediated glucose uptake [2]. 

Because of these pieces of evidence, Mg supplementation has been suggested as a possible non-pharmacologic, economic and safe treatment for the prevention and the metabolic control of T2DM. However, prospective trials concerning the effects of Mg supplementation in people with or at risk of T2DM are limited [112,113]. A modest beneficial effect of Mg supplements on glycemic profiles have been found in many, but not all, studies. A systematic review and meta-analysis including 18 double-blind randomized controlled trials (12 in subjects with diabetes and six in subjects at elevated risk of T2DM) reported that Mg supplementation may have some beneficial actions improving glucose parameters in people with T2DM and to improve insulin-sensitivity parameters in subjects at high risk of T2DM [114]. Using an umbrella review to map and grade health outcomes linked to Mg intake and supplementation, our group recently confirmed that an elevated Mg intake is associated with a decreased risk of T2DM [86]. 

## 11. Mg in Cardiometabolic Syndrome

There are also convincing proofs of a link of Mg deficit with metabolic syndrome [2,6,115]. In epidemiological studies, dietary Mg inadequacy has been connected with an increased risk of glucose intolerance, metabolic syndrome, and T2DM [115,116,117]. Intracellular Mg deficit, causing a defective activity of all the Mg-dependent kinases involved in the insulin signaling, and increasing oxidative stress would favor insulin resistance and resulting metabolic conditions, including glucose intolerance, metabolic syndrome and T2DM.

Mg-deprivation in sheep caused an impairment of insulin-mediated glucose uptake [118], while Mg supplementation delayed the development of the disease in a rat model of diabetes [119]. Lower fasting insulin levels were found in healthy women without diabetes with higher Mg intakes [120]. Total dietary Mg intakes were inversely related to insulin responses to an oral glucose tolerance test [121]. 

## 12. Mg and Asthma and Respiratory Insufficiency

First, Haury in 1940 proposed a role for Mg in asthma showing a beneficial clinical response after i.v. Mg sulfate administration in two hospitalized patients having acute exacerbations of asthma [122]. In the following decades, other reports confirmed positive results of Mg i.v. treatment in acute airway constriction, suggesting a possible beneficial action of Mg in the mechanism of bronchial dilation [123,124], although other reports did not confirm the therapeutic effect [125,126]. Administration of i.v. Mg seems to increase, in an additive fashion, the bronchial dilating effect of terbutaline [127] and salbutamol [128] in improving functional pulmonary tests.

Mg modulates the contractile state of bronchial smooth muscle cells; Mg depletion triggers bronchial contraction and spasm, while Mg restoration produces bronchial relaxation. Several possible mechanisms have been postulated for the positive Mg action to relax bronchial smooth muscle, such as the Ca channel blocking action of Mg [69], a decreased sensibility to the depolarizing action of acetylcholine [92], a stabilization of mast cells and T-lymphocytes [129], and a stimulation of nitric oxide [130] and prostacyclin [131]. In the general population, significant positive independent associations of dietary Mg intake with lung function and inverse associations with airway reactivity, inhaled methacholine, and respiratory symptoms (wheezing) have been reported, suggesting that a low Mg intake may be implicated in the etiology of asthma [132].

However, total Mg serum levels are not clinically useful, since no differences were found in serum Mg in patients with asthma during acute exacerbation compared to a non-asthmatic population and serum total Mg is not predictive of the severity of the asthmatic attacks, or of the bronchial dilating response to Mg infusion [133]. Conversely, cellular Mg (more related to body Mg status) was found to be reduced in asthmatic subjects when compared to non-asthmatic controls [134]. In addition, our group demonstrated a direct correlation, in asthmatic patients, between cellular Mg levels and the methacholine bronchial reactivity confirming the presence of intracellular Mg alterations in asthma and have proposed an additive role for a non-pharmacological Mg supplementation in asthmatic patients [135]. 

Altogether, the available data suggest a role for cellular and body Mg deficit as a modulator of smooth muscle bronchial reactivity and contractility, facilitating bronchoconstriction in predisposed asthmatic subjects, and a possible preventive and/or therapeutic role for additive use of Mg administration in these persons [136]. 

## 13. Mg and Psychiatric Disorders

Several psychiatric disorders including anxiety, depression, irritability, insomnia, hypochondriasis, panic attacks, hyperexcitability, headache, dizziness, tremors, and psychotic behavior have been associated with Mg deficiency. Neuromuscular symptoms may be associated, including asthenia, muscular weakness, and myalgias (e.g., chronic fatigue syndrome and fibromyalgia) [137].

A number of enzymes and cellular reactions involved in stress responses are Mg-dependent [15]. Serum Mg levels have been proposed to be reduced in subjects with depression [138]. A recent study by Noah et al. showed that nearly half (forty-four percent) of patients screened for stress had a latent Mg insufficiency [139]. 

Mg deficit may produce electrophysiological evidence of hyperexcitability in the central nervous system (CNS). In Mg deficient rats, electroencephalogram (EEG) modifications were monitored during auditory stimuli. Several alterations with spike activity were found in the EEG, suggesting that auditory stimulation induced behavioral changes in Mg-deficient rats, which may be linked to Mg related-increased excitability of the CNS [140]. 

In humans, Mg insufficiency has been connected with neuro-muscular hyperexcitability [141]. Various possible mechanisms may link Mg deficiency to nervous hyperexcitability, such as the previously described Mg modulatory actions on cellular Ca, the increased peroxidation, the hyper-activation of some excitatory neurotransmitters, (i.e., acetylcholine, catecholamines, NMDA and non-NMDA receptors of excitatory aminoacids), and a decreased activity of inhibitory neurotransmitters (i.e., gamma-aminobutyric acid (GABA), taurine, glutaurine, adenosine), as well as an increased production of neuropeptides, inflammatory cytokines, prostanoids, and a decreased activity of anti-oxidant defenses [137]. 

In relation to these links with the transduction and biological pathways implicated in depression, and because of the modulatory role of Mg on the ion channel of the NMDA-receptor complex [142], Mg supplements have been proposed to be helpful in the treatment of depression [143,144]. Some antidepressant drugs such as sertraline and amitriptyline have been suggested to increase intracellular Mg levels [145]. A systematic review showed that higher Mg intakes were associated with reduced depression symptoms [144]. Oral Mg supplements may provide advantage in the prevention of depressive symptoms and may be supportive as an adjunctive therapy. The effect of Mg supplementation on stress and anxiety is less documented. However, more interventional and prospective studies are needed in order to establish a clear role for Mg supplementation as a possible adjunct care in the treatment of depression, and other psychiatric disorders.

Mg has also been suggested as an adjuvant treatment in the therapy of insomnia. Mg, in addition to being a natural NMDA antagonist and a GABA agonist, also has a relaxant action, and may increase melatonin levels, thus helping to improve sleep [146]. 

## 14. Mg and Cognitive Decline

A possible protective action of Mg in cognitive deterioration and AD was already suggested in 1990 [147]. Mg ion is important for a normal neuronal maturation and is present in the cerebrospinal fluid (CSF) in the CNS [148]. Mg passes the blood–brain barrier, and is actively transported by choroidal epithelial cells into the CSF [148].

Alterations of Mg metabolism are present in patients with dementia: total and ionized serum Mg levels, and various tissues Mg content have all consistently be found reduced in patients with AD [149,150,151,152]. 

Mg concentrations in the brain affect multiple biochemical processes involved in cognitive functions, including cell membrane stability and integrity, NMDA-receptor response to excitatory stimuli, and Ca-antagonist action [19]. It has been suggested that the neurotoxic effect of some metals, such as aluminum, may be related to an alteration of the incorporation of Mg into brain neurons, thus impairing Mg protective effects on brain tissue [147]. Mg has been reported to expedite toxin clearance, reduce neuroinflammation, inhibit the pathologic processing of amyloid protein precursor, inhibit abnormal tau protein phosphorylation, and reverse deregulation of NMDA receptors. However, the mechanisms of these effects are not completely clear [153]. 

Mg-L-threonate administration has been reported to reduce neuroinflammation and decrease beta-amyloid deposition in experimental models of AD [154,155], and to enhance learning abilities, working and short- and long-term memory in rats [156]. Experimental animal studies are promising and may suggest that Mg supplementation if started at the early stages of cognitive deficits may decrease the slope of memory fall and cognitive decline [157].

In humans, only a few clinical trials have studied the role of Mg in cognitive health. Epidemiologically, it has been suggested that people consuming diets rich in Mg may have a reduced risk of cognitive decline. In 1400 healthy adult men followed for eight years, elevated dietary Mg intakes were associated with a reduced risk of developing mild cognitive impairment [158]. In another cohort study including more than 1000 community-dwelling Japanese participants aged over 60 years and followed for 17 years, it was found that those who were assuming more than 200 mg/day of Mg had thirty-seven percent less chances to develop any type of dementia and seventy four percent less chances to develop vascular dementia [159]. A short-term (12 weeks) randomized controlled trial suggested that Mg may help in improving cognitive abilities in elderly subjects with memory complaints [160]. Long-term prospective randomized clinical trials with Mg supplementation are needed to confirm if Mg-rich diets may help in preventing dementia and/or cognitive impairment.

## 15. Mg and Osteoporosis

Dietary Mg deficit has been hypothesized as a potential risk factor for osteoporotic disease and bone loss. Epidemiologic studies have shown that elevated dietary intakes of Mg were positively and significantly related to bone mineral density (BMD). On the opposite, inadequate dietary Mg intakes were linked to an increased rate of bone loss in postmenopausal osteoporotic women [161,162]. In the Health, Aging and Body Composition Study, it was observed that higher Mg intakes were associated with higher BMD in healthy white participants, aged 70 to 79 years at baseline [163]. Following a selective dietary Mg deprivation, Mg-depleted mice with frank hypomagnesemia developed osteoporosis, increased skeletal fragility associated with increased bone resorption, decreased bone formation, and impaired bone growth [164,165]. Elevated concentrations of inflammatory cytokines may play a role in explaining these bone alterations, although a clear pathophysiologic link remains undefined. Rude and Gruber showed that an increased osteoclastic bone resorption was associated with increased levels of inflammatory substance P and TNF-alfa in bone from Mg-deficient rats [166].

In addition, Mg is necessary for vitamin D synthesis, transport, and activation; hence, Mg deficits would impair the production of the active form of vitamin D, 1,25-OH2 D3, and cause a resistance to PTH and vitamin D actions [167]. The effects of Mg deficiency added together with an altered PTH responsiveness and low 1,25-OH2 D3 synthesis would impair the bone formation and mineralization processes and would reduce the quality, and strength of the bone as well as the BMD. It has been hypothesized that Mg supplementation in doses sufficient to restore a normal bone turnover may reduce the bone loss and prevent the risk of osteoporosis [168,169]. 

In participants to the cohort “Osteoarthritis Initiative” followed for 8 years, it was found that women with the higher dietary Mg intake had a twenty-seven percent reduced risk for future fractures, confirming the positive role of maintaining an adequate Mg balance on the risk of osteoporosis and fragility fractures [170].

## 16. Mg and the Muscle Health

Mg ion has a key role in all enzymes utilizing or synthesizing muscle ATP, and thus in the production of muscle energy, and indirectly in the contraction and relaxation processes. Mg deficit has been related to a poor muscle performance.

Severe Mg deficits have been suggested to cause weakness, muscle pain and night cramps. It has been proposed that Mg deficit may contribute to the development of fibromyalgia [171]. Data on the effects of Mg supplements in fibromyalgia symptoms are scarce, although it was suggested that Mg supplements may be used to reduce tenderness, pain, and symptom severity in fibromyalgic subjects [172]. 

Dietary Mg deficiency in rats boosts the production of free radical in skeletal muscle and may cause several alterations in muscle cell metabolism together with structural impairments affecting the production of muscle energy needed for muscle contraction and relaxation [173].

In humans, Dominguez et al., showed a strong and independent relationship between serum Mg levels with muscle performance and several muscle parameters [174]. In young volunteers, Brilla et al. showed that Mg supplements (up to 8 mg/kg daily) were able to enhance muscle strength and endurance performance, and to reduce the oxygen consumption [175]. In older subjects, Veronese et al. showed that oral Mg supplementation (three hundred mg/day) was able to improve the physical performance, in particular in those subjects with a baseline low Mg dietary intake, proposing that Mg supplementation may help in preventing or delaying the decline in physical performance with age [176].

## 17. Mg and Cancer

In regard to cancer, Mg intake has been connected with the incidence of some cancers. However, the relation between Mg and cancer is complex, and nowadays there are more questions than answers [177]. In animal models, Mg may exert both anti- and pro-tumor effects such as inhibition of tumor growth at its primary site and facilitation of tumor implantation at its metastatic sites. In Mg-deficient mice, low Mg may both restrict and foster tumorigenesis, since inhibition of tumor growth at its primary site is observed in the face of increased metastatic colonization [177].

Oxidative stress and trace elements have been implicated in the development of breast cancer. However, how they impact the pathogenesis of the disease remain unclear [178]. Lower serum Mg levels in women with breast cancer may compromise the antioxidant defense systems involved in the carcinogenesis process. A study evaluated Mg metabolism, the superoxide dismutase activity, and its relation with oxidation stress in women with breast cancer. The authors reported that breast cancer patients display a complex alteration of Mg homeostasis, characterized by low dietary Mg intakes, reduced plasma, and erythrocytes Mg levels and an increase in Mg excretion in the urine [179].

Mg supplementation may have a protective effect on experimentally induced fibrosarcoma in rats [180], and may inhibit nickel-induced carcinogenesis in the rat kidney [181].

Mg has been suggested to have anti-tumor effects in colorectal cancer by inhibiting c-myc expression and ornithine decarboxylase activity in the mucosal epithelium of the intestine [182]. In human studies, high dietary Mg consumption has been suggested to be protective for the risk of developing colorectal cancer [183]. In postmenopausal women, it has been proposed that a higher ratio of serum Ca to Mg may increase the risk for breast cancer [184]. Ca, Mg or Ca:Mg intake ratio may interact with polymorphisms in the *SLC7A2* gene in associations with colorectal cancer [185].

Higher Mg intake was associated with a lower risk of liver cancer, based on an analysis of the National Institute of Health-American Association of Retired Persons (NIH-AARP) Diet and Health Study prospective cohort [186].

One of the reasons that the independent relationship of Mg intake and cancer protection is not easy to define is because dietary Mg content parallels fiber content and is mostly obtained from green leafy vegetables and whole cereals, rich sources of fiber, that are themselves cancer protective. 

## 18. Conclusions

A chronic Mg deficiency is frequently present in older adults. Low-grade chronic inflammation (inflammaging) is frequently present in numerous age-related chronic diseases, and with the aging process itself. Since a chronic Mg inadequacy may cause an exaggerated production of inflammatory mediators and ROS, and it may trigger an inflammatory state, our group has previously hypothesized that the chronic Mg insufficiency may be one of the mediators helping to explain the link between inflammaging and aging-related diseases [19,49] (Figure 2). It is possible to hypothesize that preserving an optimal Mg balance during the course of life may help to prevent inflammaging and related conditions associated with Mg inadequacy and may thus help to lengthen healthy life. 

However, while it is advisable to maintain a satisfactory Mg balance with a sufficient dietary intake of Mg, the possible role of Mg supplements is still unclear.

Very few long-term longitudinal blind studies on the effects of Mg supplementation have been performed. The possibility that maintaining a satisfactory Mg balance throughout life may become an economic and safe health strategy in the growing aging population is a suggestive hypothesis that needs to be proven by future prospective studies.

## Figures and Tables

**Table 1:** Characteristics of Ionic Mg.

Element category: alkaline earth metal Atomic number: 12 Atomic weight: 24.305 g/mol Valence: 2

**Table 2:** Determining factors of Mg balance.

Gastrointestinal absorption Renal excretion Diet Requirements for healthy individuals are 5–7 mg/kg of body weight/day to stay in balance Extracellular Mg is in equilibrium with Mg in the storages Bone is the main storage site of Mg Decreased tubular reabsorption, osmotic diuresis or drugs may cause hypomagnesemia, by Mg wasting

**Table 3:** Mechanisms of Mg insufficiency in the elderly.

**Primary Mg Deficiency:**
Insufficient Mg dietary intake Reduced Mg absorption (often in parallel with reduced vitamin D levels) Increased urinary Mg excretion (often related to reduced kidney function and tubular reabsorption, which are common in old age)
**Secondary Mg Deficiency:**
Linked to age-related diseases and comorbidities Linked to drug action causing Mg loss in the urine (i.e., diuretics, proton pump inhibitors)

## Data Availability

No new data were created or analyzed in this study. Data sharing is not applicable to this article.
